# Comparative effectiveness of telerehabilitation vs. face-to-face rehabilitation in adults with chronic low back pain: a systematic review and meta-analysis of randomized controlled trials

**DOI:** 10.3389/fpubh.2026.1965765

**Published:** 2026-09-30

**Authors:** Shaowen Liu, Jiayin Yang, Beibei Lei, Yage Wang, Weisheng Zhuang, Hongjian Liu

**Affiliations:** 1 Rehabilitation Medicine College, Henan University of Chinese Medicine, Zhengzhou, China; 2 Department of Rehabilitation, Henan Provincial People's Hospital, School of Rehabilitation Medicine, Henan University of Chinese Medicine, Zhengzhou, China; 3 Department of Otorhinolaryngology, Head and Neck Surgery, Henan Provincial People's Hospital, People's Hospital of Zhengzhou University, People's Hospital of Henan University, Zhengzhou, Henan, China

**Keywords:** digital health, disability, exercise therapy, pain intensity, physical function

## Abstract

**Background:**

Chronic low back pain (CLBP) is a major cause of disability worldwide and creates substantial rehabilitation demand. When core rehabilitation content is similar, the comparative effectiveness of telerehabilitation (TR) and face-to-face rehabilitation as delivery modes remains unclear.

**Methods:**

Methods: This meta-analysis used strict eligibility criteria to distinguish the two delivery pathways and better isolate delivery-mode effects. PubMed, Web of Science, the Cochrane Library, and Embase were searched for randomized controlled trials in adults with CLBP. Paper-based home exercise programs, continuous hybrid delivery, and remote-assessment-only designs were excluded. Random-effects meta-analyses used standardized mean differences (SMDs) with 95% confidence intervals (CIs). RoB 2 and GRADE assessed risk of bias and evidence certainty. Sensitivity and subgroup analyses were conducted.

**Results:**

Ten trials involving 562 participants were included. Content-matched primary analyses found no significant difference between TR and face-to-face rehabilitation in pain intensity (7 trials; SMD = −0.05, 95% CI − 0.34 to 0.24; very low certainty) or functional disability (8 trials; SMD = 0.22, 95% CI − 0.07 to 0.52; low certainty). In secondary analyses including two trials that provided manual therapy only to the face-to-face group, pain intensity did not differ significantly (9 trials; SMD = −0.01, 95% CI − 0.26 to 0.24), whereas functional disability favored face-to-face rehabilitation (10 trials; SMD = 0.26, 95% CI 0.01 to 0.52). Subgroup interaction tests were nonsignificant for intervention duration, weekly treatment frequency, and supervision status.

**Conclusion:**

When rehabilitation content was similar, TR and face-to-face rehabilitation showed no statistically significant differences in pain intensity or functional disability. However, these findings should not be interpreted as evidence of equivalent effectiveness between TR and face-to-face rehabilitation, because the certainty of evidence was low for functional disability and very low for pain intensity, and the functional disability estimate was sensitive to the exclusion of one study. Future high-quality trials with comparable interventions and longer follow-up are needed, particularly direct comparisons of supervised and unsupervised TR.

**Systematic review registration:**

PROSPERO, CRD420261467122.

## 1 Introduction

Chronic low back pain (CLBP) is generally defined as pain, with or without muscle tension, located between the costal margin and the gluteal folds and persisting for more than 12 weeks (1). The global burden of CLBP continues to increase, largely because of population aging and growth (2). Face-to-face rehabilitation enables direct therapist supervision and individualized treatment adjustments. It may also include adjunct interventions such as manual therapy. This approach therefore remains a mainstay of clinical management (3–6). However, time demands, mobility limitations, and financial costs may restrict access to care and reduce long-term treatment adherence (7, 8). These barriers have increased interest in alternative delivery models that may maintain clinical effectiveness while improving accessibility. The COVID-19 pandemic accelerated the adoption of telemedicine, including telerehabilitation (TR). TR may offer greater logistical flexibility, reduce geographic barriers, and potentially lower care-related costs. Accordingly, TR has been increasingly integrated into the management of musculoskeletal conditions (7, 9, 10).

Although previous evidence suggests that TR may be beneficial for musculoskeletal conditions, its comparative effectiveness relative to face-to-face rehabilitation in patients with CLBP remains uncertain (11). A previous meta-analysis suggested that TR may be a viable intervention for musculoskeletal disorders. However, only four included trials involved low back pain, limiting the applicability of its findings to CLBP (12). A more recent meta-analysis comparing TR with traditional care included trials in which the control intervention was not delivered exclusively face to face. It also included trials involving paper-based home exercise programs or remote assessment without remotely delivered active rehabilitation. These differences in intervention and comparator definitions blurred the distinction between the two delivery pathways. The limited number of eligible trials also restricted meaningful subgroup analyses (13). Consequently, it remains unclear whether any observed differences reflect the mode of delivery itself or differences in intervention content, therapist contact, or adjunct treatment. Because the primary contrast of interest is the rehabilitation delivery pathway, broad eligibility criteria may reduce comparability across trials and complicate the interpretation of pooled estimates. A systematic review using more clearly defined intervention and comparator criteria is therefore warranted.

To address this gap, this systematic review and meta-analysis applied stringent eligibility criteria to exclude paper-based home exercise programs, continuous hybrid delivery models, and studies involving remote assessment without remotely delivered active rehabilitation. The primary analysis was restricted to trials in which the core rehabilitation components were broadly comparable between groups. A broader secondary analysis included all otherwise eligible trials to examine whether asymmetric adjunct interventions influenced the pooled estimates. This analytical framework was designed to distinguish differences associated with the delivery pathway from those associated with intervention composition and to provide a more interpretable comparison of TR and face-to-face rehabilitation for CLBP.

## 2 Materials and methods

### 2.1 Study design and registration

This systematic review and meta-analysis was conducted according to a prospectively registered protocol and reported in accordance with the Preferred Reporting Items for Systematic Reviews and Meta-Analyses 2020 statement (PRISMA 2020) (14). The protocol was prospectively registered in the International Prospective Register of Systematic Reviews (PROSPERO; registration number CRD420261467122).

### 2.2 Search strategy

A systematic literature search was conducted in PubMed, the Web of Science Core Collection, the Cochrane Central Register of Controlled Trials (CENTRAL), and Embase. The search process was conducted in two stages: an initial search was performed during protocol development, followed by a subsequent search before data extraction and manuscript preparation to identify additional eligible studies. The final search covered each database from inception to 2 July 2026. The search strategies combined database-specific controlled vocabulary and free-text terms related to CLBP, telerehabilitation, and randomized controlled trials. The principal search concepts were low back pain and telerehabilitation, with relevant synonyms and spelling variants adapted for each database. No language restrictions were applied. The full search strategy for each database, including the search terms and Boolean operators, is provided in Supplementary material 1.

### 2.3 Inclusion and exclusion criteria

Study eligibility was defined according to the PICOS framework:

Population: Adults aged 18 years or older with CLBP, defined as low back pain persisting for more than 12 weeks.

Intervention: Active telerehabilitation delivered primarily through remote communication technologies, including mobile applications, videoconferencing, or telephone-based delivery.

Comparator: Face-to-face rehabilitation in which all core active rehabilitation sessions were delivered in person throughout the intervention period.

Outcomes: The primary outcomes were pain intensity, assessed using the Visual Analog Scale (VAS), Numeric Rating Scale (NRS), Numeric Pain Rating Scale (NPRS), Verbal Numeric Rating Scale (VNRS), or Quantitative Visual Analog Scale (QVAS), and functional disability, assessed using the Oswestry Disability Index (ODI) or the Roland–Morris Disability Questionnaire (RMDQ).

Study design: Randomized controlled trials (RCTs).An in-person baseline assessment or a single initial technical orientation session was permitted in the TR group, provided that the core active rehabilitation program was subsequently delivered remotely. Eligible studies were required to report at least one primary outcome at or immediately after completion of the intervention.

Studies were excluded if (1) the intervention consisted of a paper-based home exercise program without remotely delivered active rehabilitation; (2) the intervention used a continuous hybrid model in which remote and face-to-face rehabilitation were combined throughout the treatment period; (3) the remote component was limited to education, symptom monitoring, reminders, or assessment without active rehabilitation; or (4) the study was not randomized.

All eligible studies were included in the systematic review. For each outcome, all eligible trials reporting that outcome were included in the broader secondary meta-analysis. For the content-matched primary analysis, the core rehabilitation intervention was required to be broadly comparable between groups in terms of intervention content, treatment frequency, dose, and duration. Differences inherent to the rehabilitation delivery pathway, including supervision format, therapist contact, and the mode of intervention delivery, were not considered criteria for exclusion because these components represent essential characteristics distinguishing telerehabilitation from face-to-face rehabilitation. In addition, no additional therapeutic intervention intended to affect pain or functional disability could be provided exclusively to one group. Trials in which adjunct manual therapy was provided exclusively to the face-to-face group were therefore included in the broader secondary analysis but excluded from the content-matched primary analysis.

### 2.4 Study selection

Two reviewers independently screened all retrieved records. Duplicate records were removed before screening. The reviewers independently screened titles and abstracts to identify potentially eligible reports. The full texts of potentially eligible reports were then assessed independently against the eligibility criteria. Disagreements were resolved through discussion, with adjudication by a third reviewer when consensus could not be reached. Reasons for exclusion at the full-text stage were recorded, and the study-selection process was summarized in a PRISMA flow diagram.

### 2.5 Data extraction

Two reviewers independently extracted data using a standardized data-extraction form. Disagreements were resolved through discussion, with adjudication by a third reviewer when consensus could not be reached. The extracted data included the first author, publication year, country or region, sample size in each group, participant characteristics (sex distribution, age, and duration of LBP), intervention and comparator characteristics (intervention content, treatment frequency, total intervention duration, and supervision format), outcome measures, and the means and standard deviations (SDs) of change-from-baseline scores at the end of the intervention. If change scores were unavailable, the corresponding baseline and post-intervention means, SDs, and group sample sizes were extracted. For studies reporting multiple assessment time points, the assessment conducted at or closest to completion of the intervention was selected for the primary meta-analysis. Longer-term follow-up data were not included in the primary quantitative synthesis.

### 2.6 Risk of bias assessment

Two reviewers independently assessed risk of bias using the revised Cochrane risk-of-bias tool for randomized trials (RoB 2), specifically the version for individually randomized parallel-group trials. The effect of assignment to intervention was selected as the effect of interest. Separate RoB 2 assessments were conducted for pain intensity and functional disability at the assessment conducted at or closest to completion of the intervention. The assessment covered bias arising from the randomization process, bias due to deviations from intended interventions, bias due to missing outcome data, bias in outcome measurement, and bias in the selection of the reported result. Domain-level and overall risk-of-bias judgments were classified as low risk, some concerns, or high risk. Disagreements were resolved through discussion between the two reviewers, with adjudication by a third reviewer when consensus could not be reached.

### 2.7 Statistical analysis

Data synthesis and statistical analyses were conducted using Review Manager version 5.4.1 and Stata version 15.0. Because the included studies assessed pain intensity and functional disability using different measurement instruments, pooled effects were expressed as standardized mean differences (SMDs) with 95% confidence intervals (CIs). Mean differences (MDs) were not pooled because the included trials did not use a common measurement scale for either outcome. All scales were oriented in the same direction before pooling, with lower scores indicating better outcomes. Accordingly, negative SMDs favored TR, whereas positive SMDs favored face-to-face rehabilitation. The meta-analyses were based on mean changes from baseline and their corresponding standard deviations (SDs). When change scores were not reported, mean changes were calculated using Equation 1, and the corresponding SDs were estimated using Equation 2. In accordance with the recommendations of the Cochrane Handbook, a baseline-to-post-intervention correlation coefficient of r = 0.50 was assumed when the correlation was not reported (15). For studies reporting means and corresponding 95% CIs but not SDs, SDs were derived from the CIs and group sample sizes using Equation 3, in accordance with the Cochrane Handbook (15). For studies reporting medians and interquartile ranges (IQRs), means and SDs were estimated using the methods proposed by Wan et al. (16), with Equation 4 used to estimate the mean and Equation 5 used to estimate the SD. To assess the influence of data conversion, a sensitivity analysis was conducted after excluding studies in which means or SDs were estimated from reported 95% CIs, medians, or IQRs. Studies in which change scores and their SDs were calculated from reported baseline and post-intervention data were retained in this sensitivity analysis. The equations used for these calculations are presented below:


$${\overset{-}{X}}_{\text{change}}={\overset{-}{X}}_{\text{final}}-{\overset{-}{X}}_{\text{baseline}}$$ (1)

$${\mathrm{SD}}_{\text{change}}=\sqrt{{\mathrm{SD}}_{\text{baseline}}^{2}+{\mathrm{SD}}_{\text{final}}^{2}-2r\cdot {\mathrm{SD}}_{\text{baseline}}\cdot {\mathrm{SD}}_{\text{final}}}.$$ (2)

$$\mathrm{SD}=\sqrt{n}\cdot \frac{U-L}{2t_{0.975,n-1}}$$ (3)

$$\overset{-}{X}\approx \frac{Q_{1}+m+Q_{3}}{3}$$ (4)

$$\begin{array}{c} \mathrm{SD}\approx \frac{Q_{3}-Q_{1}}{2\Phi^{-1}(\frac{0.75n-0.125}{n+0.25})} \end{array}.$$ (5)

In these formulas, *X̄* represents the mean; *SD* represents the standard deviation; n represents the sample size; *L* and *U* represent the lower and upper limits of the *95% CI*, respectively; m represents the median; *Q1* and *Q3* represent the first and third quartiles, respectively; r represents the correlation coefficient between baseline and post-intervention values; and *Φ^−1^* represents the inverse cumulative distribution function of the standard normal distribution.

When at least 10 studies were available for an outcome, funnel plots were visually examined for asymmetry and potential small-study effects. Because funnel plot asymmetry may arise from clinical heterogeneity, methodological heterogeneity, reporting bias, or chance, the findings were interpreted as exploratory rather than definitive evidence of publication bias. Leave-one-out sensitivity analyses were conducted for each pooled outcome to assess the influence of individual studies on the summary effect estimate. A random-effects model was specified *a priori* for all meta-analyses because clinical and methodological heterogeneity was anticipated across studies.

Exploratory subgroup analyses were conducted within the content-matched primary analysis set to examine potential sources of heterogeneity. Studies were stratified according to intervention duration (4 vs. 8 weeks), weekly intervention frequency (three sessions per week vs. two sessions per week), and supervision status (supervised vs. unsupervised). Subgroup analyses were performed only when each subgroup included at least three studies. Differences between subgroups were evaluated using formal tests for subgroup interaction.

### 2.8 Certainty of evidence assessment

The certainty of the evidence for each primary outcome in the content-matched primary analysis at the end of the intervention was assessed using the Grading of Recommendations Assessment, Development and Evaluation (GRADE) approach. Two reviewers independently assessed five domains: risk of bias, inconsistency, indirectness, imprecision, and publication bias. Evidence from randomized trials was initially rated as high certainty and was downgraded by one or two levels for serious or very serious concerns in any GRADE domain. Risk of bias was assessed on the basis of the RoB 2 judgments for the studies contributing to each pooled estimate, with consideration of their relative contribution to the result. Inconsistency was assessed by considering the magnitude and direction of the individual study effects and the extent of unexplained statistical heterogeneity. Indirectness was assessed by comparing the populations, interventions, comparators, outcomes, and assessment time points of the included studies with the review question. Imprecision was assessed by considering the width of the 95% CI, whether the interval included both no effect and potentially important benefit or harm, and the total information size. Publication bias was considered on the basis of funnel-plot asymmetry when at least 10 studies were available, together with evidence of small-study effects or selective non-publication. The overall certainty of the evidence for each outcome was rated as high, moderate, low, or very low. Disagreements were resolved through discussion, with adjudication by a third reviewer when consensus could not be reached. Reasons for downgrading were reported in the footnotes to the Summary of Findings table.

## 3 Results

### 3.1 Literature search results

The database searches yielded 863 records. After 72 duplicate records were removed, 791 records underwent title and abstract screening. Following full-text assessment, 10 randomized controlled trials met the eligibility criteria. Studies excluded after full-text assessment and the reasons for exclusion are provided in Supplementary material 2. The study-selection process is presented in the PRISMA flow diagram in Figure 1.

**Figure 1:**
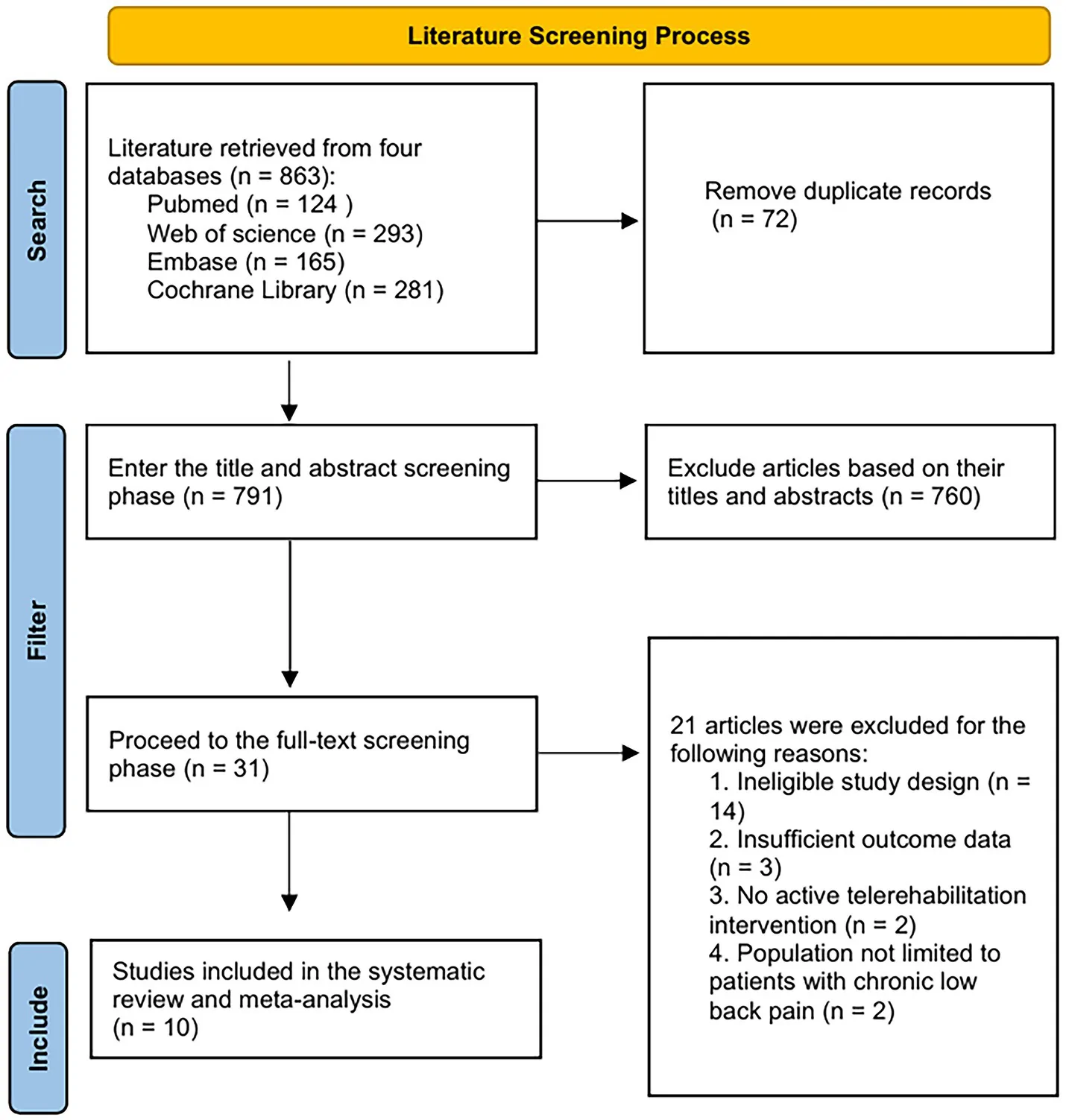
Flow diagram of the literature search and study selection process.

### 3.2 Study characteristics

These 10 trials (17–26) enrolled a total of 562 patients with CLBP, including 255 men (45.4%) and 307 women (54.6%). Mean participant ages ranged from 21 to 61 years. The trials were conducted in Nigeria, Türkiye, South Korea, Spain, China, Iran, and Saudi Arabia. The rehabilitation programs included McKenzie-based exercises, core stabilization exercises, strengthening and stretching exercises, and multicomponent programs that incorporated health education. Telerehabilitation was delivered through various modalities, including mobile applications, telephone-based communication, asynchronous video programs, and real-time videoconferencing. In eight trials, the core rehabilitation components were broadly comparable between the TR and face-to-face groups. In the remaining two trials, adjunct manual therapy was provided exclusively to the face-to-face group (18, 24). The eight content-matched trials were included in the primary analysis, whereas all otherwise eligible trials reporting the outcome of interest were included in the broader secondary analysis. Detailed study and intervention characteristics are presented in Supplementary material 3.

### 3.3 Risk of bias assessment results

For trials reporting both primary outcomes, the domain-level and overall risk-of-bias judgments were identical for pain intensity and functional disability. The assessments for the two outcomes were therefore presented separately in the summary table but are summarized together at the study level in the traffic-light plot. One trial reported functional disability but not pain intensity and was therefore assessed only for the functional disability outcome (17). Overall, 40% of the included studies were judged as having some concerns, whereas 60% were judged to be at high risk of bias; none were judged to be at low risk of bias overall. The high-risk and some-concerns judgments were primarily attributable to insufficient information about allocation concealment, baseline imbalances suggestive of possible problems with the randomization process, deviations from the intended interventions, concerns arising from unblinded self-reported outcome measurement, and insufficient information on prespecified outcome analyses. At the domain level, for the randomization process, 60% of studies were judged to be at low risk of bias, 30% as having some concerns, and 10% at high risk of bias. For deviations from intended interventions, 80% of studies were judged as having some concerns and 20% at high risk of bias. Most studies were judged to be at low risk of bias due to missing outcome data (90%), with some concerns identified for one trial in this domain (10%). For measurement of the outcome, 40% of studies were judged to be at low risk of bias, 20% as having some concerns, and 40% at high risk of bias. For selection of the reported result, 70% of studies were judged as having some concerns and 30% at high risk of bias (Table 1; Figure 2). The overall risk-of-bias summary across domains is presented in Figure 3.

**Table 1:** Outcome-specific RoB 2 judgments across included studies.

Study ID	Outcome	D1	D2	D3	D4	D5	Overall
Mbada et al. (26)	Pain intensity Functional disability	Low	SC	Low	High	SC	High
Park et al. (19)	Pain intensity Functional disability	SC	SC	Low	Low	SC	SC
Villatoro-Luque et al. (20)	Pain intensity Functional disability	Low	SC	Low	Low	SC	SC
Mbada et al. (18)	Pain intensity Functional disability	Low	SC	Low	High	High	High
Shi et al. (21)	Pain intensity Functional disability	Low	SC	Low	SC	SC	SC
Dadarkhah et al. (22)	Pain intensity Functional disability	Low	SC	Low	SC	SC	SC
Fanuscu et al. (23)	Pain intensity Functional disability	Low	SC	SC	High	SC	High
Karaduman et al. (25)	Pain intensity Functional disability	High	SC	Low	Low	High	High
Akodu et al. (17)	Functional disability	SC	High	Low	Low	High	High
Alahmri et al. (13)	Functional disability Pain intensity	SC	High	Low	High	SC	High

D1, Randomisation process; D2, Deviations from the intended interventions; D3, Missing outcome data; D4, Measurement of the outcome; D5, Selection of the reported result; Low, low risk; SC, some concerns.

**Figure 2:**
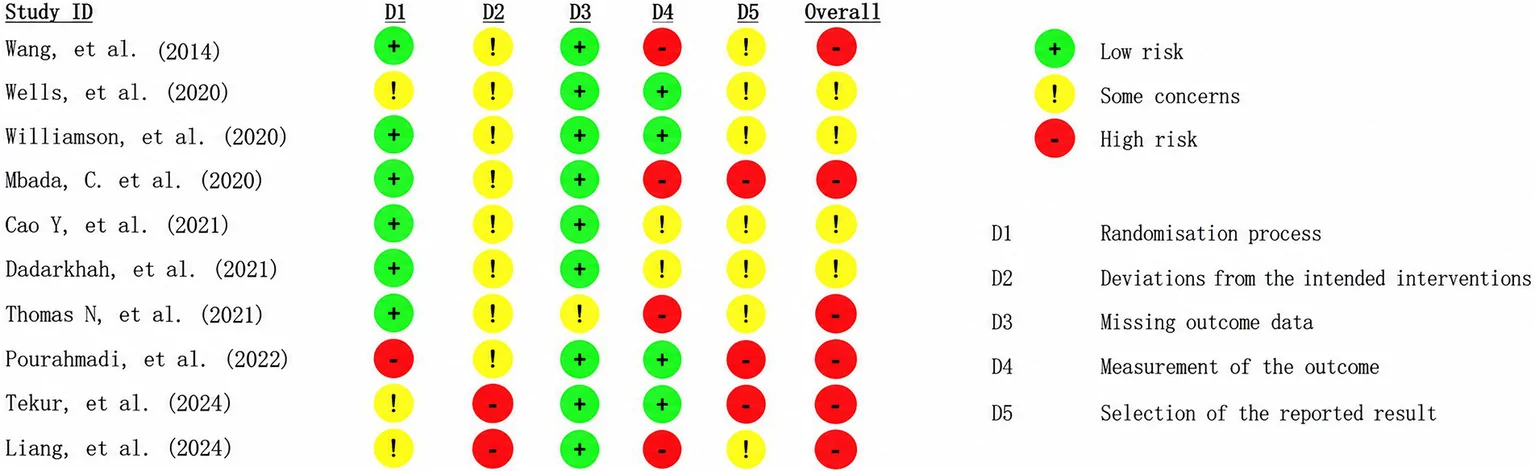
Risk of bias assessment of the included randomized controlled trials. Risk of bias was assessed using the Cochrane Risk of Bias 2 (RoB 2) tool across five domains: randomization process (D1), deviations from intended interventions (D2), missing outcome data (D3), measurement of the outcome (D4), and selection of the reported result (D5). Green, yellow, and red symbols indicate low risk of bias, some concerns, and high risk of bias, respectively.

**Figure 3:**
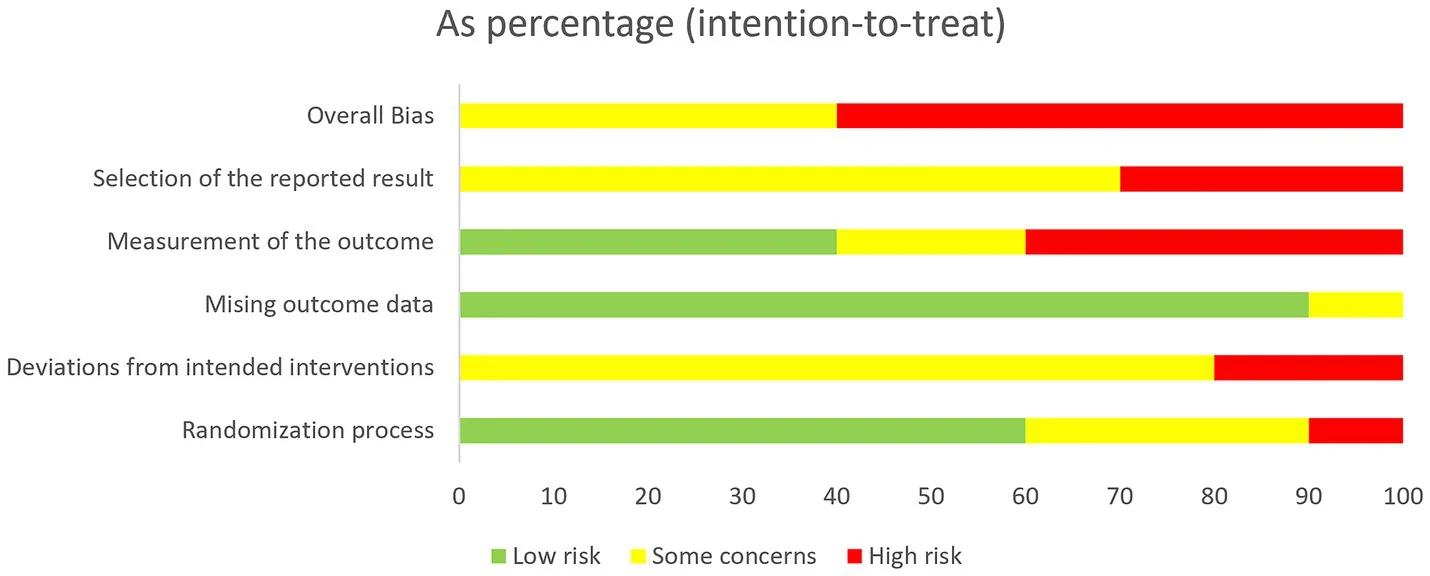
Risk-of-bias summary of the included studies. The figure presents the proportion of studies classified according to each risk-of-bias domain based on the RoB 2 assessment. Green indicates low risk of bias, yellow indicates some concerns, and red indicates high risk of bias.

### 3.4 Meta-analysis results

#### 3.4.1 Primary analysis results

##### 3.4.1.1 Pain intensity

As shown in Figure 4, the content-matched primary meta-analysis of pain intensity included 7 studies involving 414 participants, of whom 205 received TR and 209 received face-to-face rehabilitation (19–23, 25, 26). For one study, the SD was derived from the reported 95% CI and group sample size (20). The pooled analysis showed no statistically significant difference between TR and face-to-face rehabilitation (SMD = −0.05, 95% CI − 0.34 to 0.24; *p* = 0.72; *I^2^* = 54%; very low-certainty evidence). The certainty of the evidence was rated as very low because of concerns regarding risk of bias, inconsistency, and imprecision (Supplementary material 4). Exploratory subgroup analyses did not identify evidence of effect modification by intervention duration, weekly intervention frequency, or supervision status; all tests for subgroup interaction were nonsignificant (all *p* > 0.05; Table 2), indicating no evidence that intervention duration, weekly frequency, or supervision status modified the comparative effect.

**Figure 4:**
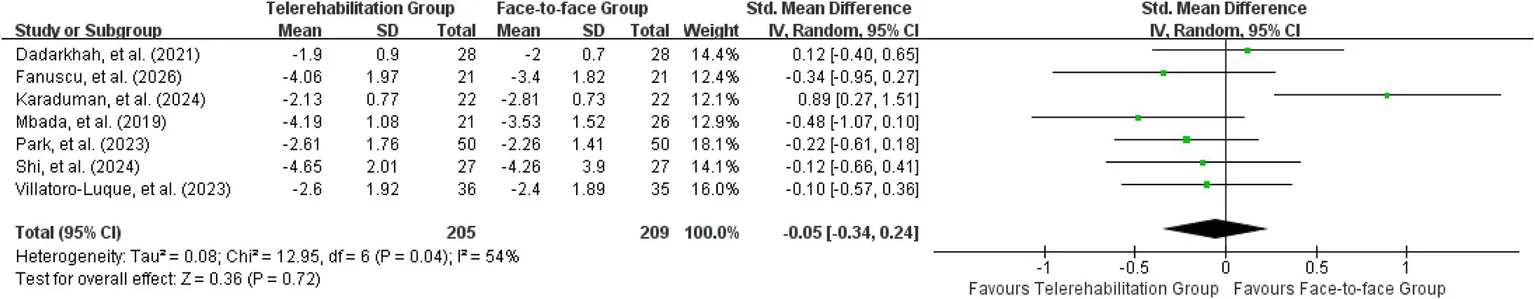
Forest plot of the primary analysis for pain intensity. The diamond represents the pooled effect estimate, and the green squares represent the effect estimates for individual studies.

**Table 2:** Summary of all meta-analysis results.

Outcome	N	Std. Mean difference (95% CI)	*p*	*I^2^* (*p*)
Pain intensity	7	−0.05[−0.34, 0.24]	0.72	54%(0.04)
(4 weeks)	3	0.23 [−0.38, 0.84]	0.47	77%(0.01)
(8 weeks)	4	−0.24[−0.51, 0.03]	0.09	0%(0.73)
(Supervised)	4	0.09 [−0.44, 0.62]	0.74	72%(0.01)
(Unsupervised)	3	−0.20[−0.47, 0.07]	0.14	0%(0.83)
Functional limitation	8	0.22 [−0.07, 0.52]	0.14	60%(0.01)
(4 weeks)	3	0.51 [−0.13, 1.14]	0.12	78%(0.01)
(8 weeks)	5	0.05 [−0.20, 0.30]	0.69	8%(0.36)
(Supervised)	5	0.20 [−0.31, 0.71]	0.45	75%(0.003)
(Unsupervised)	3	0.29 [0.02, 0.56]	0.03	0%(0.67)
2 times/week	3	0.03[−0.26,0.33]	0.83	0%(0.86)
3 times/week	5	0.35[−0.11,0.82]	0.14	73%(0.005)

##### 3.4.1.2 Functional disability

As shown in Figure 5, the content-matched primary meta-analysis of functional disability included 8 studies involving 464 participants, of whom 230 received TR and 234 received face-to-face rehabilitation (17, 19–23, 25, 26). For one study, the SD was derived from the reported 95% CI and group sample size (20). For two studies, means and SDs were estimated from the reported medians and IQRs (17, 23). The pooled analysis showed no statistically significant difference between TR and face-to-face rehabilitation (SMD = 0.22, 95% CI − 0.07 to 0.52; *p* = 0.14; *I^2^* = 60%; low-certainty evidence). The leave-one-out sensitivity analysis indicated that the pooled estimate was sensitive to the exclusion of individual studies and should therefore be interpreted cautiously (Supplementary material 5). Subgroup analyses did not identify statistically significant subgroup differences according to weekly intervention frequency or intervention duration. Within the unsupervised subgroup, the pooled estimate favored face-to-face rehabilitation (SMD = 0.29, 95% CI 0.02 to 0.56; *p* = 0.03), whereas no statistically significant difference was observed within the supervised subgroup (SMD = 0.20, 95% CI − 0.31 to 0.71; *p* = 0.45). However, the test for interaction between the supervised and unsupervised subgroups was not statistically significant (χ^2^ = 0.10, df = 1, *p* = 0.75; *I^2^* = 0%; Table 2), indicating no evidence that supervision status modified the comparative effect.

**Figure 5:**
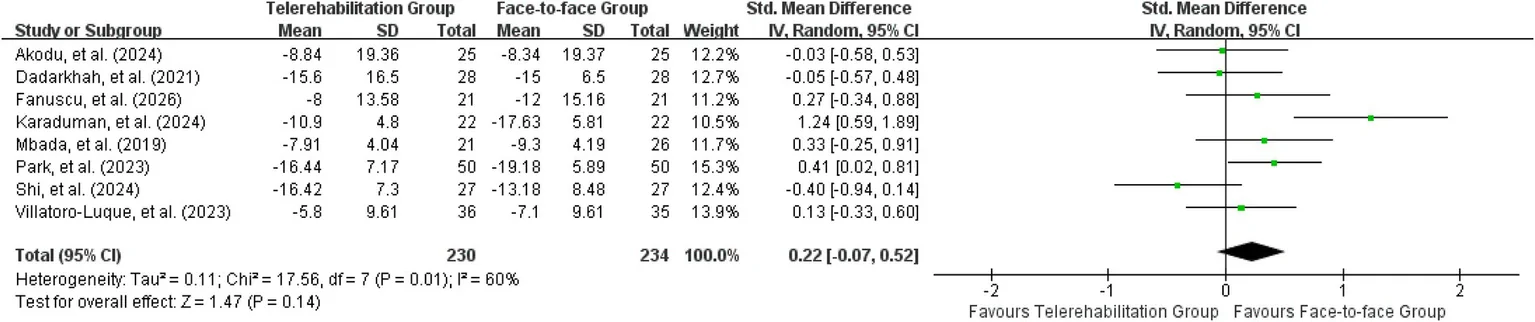
Forest plot of the primary analysis for functional disability. The diamond represents the pooled effect estimate, and the green squares represent the effect estimates for individual studies.

#### 3.4.2 Secondary analysis results

##### 3.4.2.1 Pain intensity

As shown in Figure 6, the broader secondary meta-analysis of pain intensity included 9 studies, including two trials in which adjunct manual therapy was provided exclusively to the face-to-face group. The analysis involved 532 participants, of whom 257 received TR and 275 received face-to-face rehabilitation (18–26). The pooled analysis showed no statistically significant difference between TR and face-to-face rehabilitation (SMD = −0.01, 95% CI − 0.26 to 0.24; *p* = 0.92; *I^2^* = 50%).

**Figure 6:**
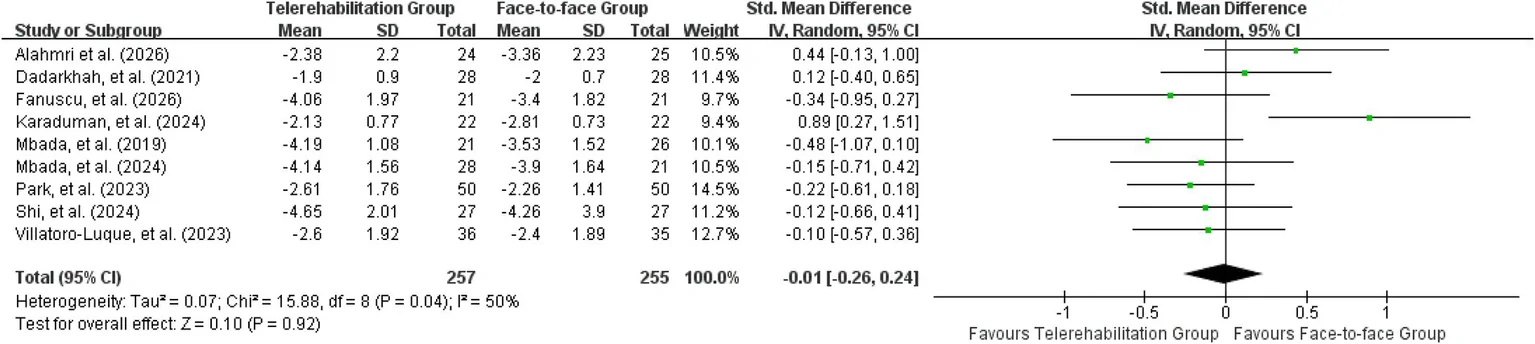
Forest plot of the secondary analysis for pain intensity. The diamond represents the pooled effect estimate, and the green squares represent the effect estimates for individual studies.

##### 3.4.2.2 Functional disability

As shown in Figure 7, the pooled analysis for functional disability included 10 studies, comprising 282 participants in the TR group and 280 in the face-to-face group (17–26). The pooled analysis showed a statistically significant effect favoring face-to-face rehabilitation (SMD = 0.26, 95% CI 0.01 to 0.52; *p* = 0.04; *I^2^* = 54%).

**Figure 7:**
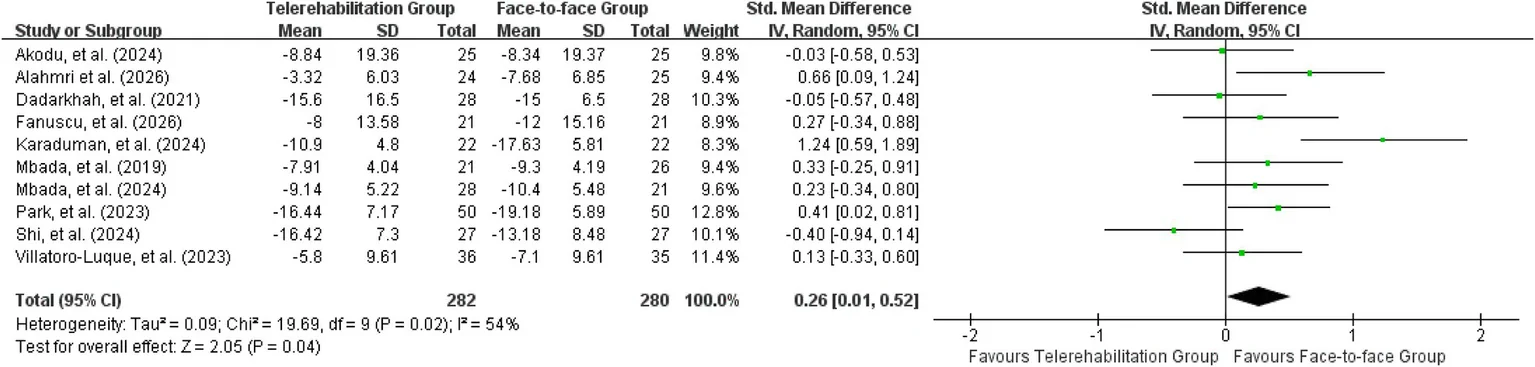
Forest plot of the secondary analysis for functional disability. The diamond represents the pooled effect estimate, and the green squares represent the effect estimates for individual studies.

### 3.5 Funnel plot assessment

Because fewer than 10 studies were included in each content-matched primary analysis, funnel plots were not generated for the primary analyses. An exploratory funnel plot was examined only for functional disability in the broader secondary analysis, which included 10 studies. The broader secondary analysis for pain intensity included only 9 studies; therefore, a funnel plot was not generated for this outcome. Visual inspection of the funnel plot for functional disability suggested some asymmetry; however, this finding was considered exploratory. Because only 10 studies were included, the observed asymmetry could reflect small-study effects, clinical heterogeneity, or chance and was not considered definitive evidence of publication bias (Supplementary material 5).

### 3.6 Sensitivity analysis results

The leave-one-out analysis for pain intensity showed that exclusion of individual studies did not result in statistically significant changes in the pooled estimate. For functional disability, exclusion of the study by Shi et al. (21) changed the pooled result from no statistically significant difference to a statistically significant effect favoring face-to-face rehabilitation (SMD = 0.31, 95% CI 0.03 to 0.59; *p* = 0.03). This finding indicated that the statistical significance of the pooled estimate was sensitive to the inclusion of this study. Excluding studies in which means or SDs were estimated from reported 95% CIs, medians, or IQRs did not materially alter the pooled estimates for either primary outcome (Supplementary material 5, showing the sensitivity results with changes in statistical significance and the analyses excluding studies requiring data estimation).

## 4 Discussion

This systematic review and meta-analysis compared the effectiveness of telerehabilitation with that of face-to-face rehabilitation for CLBP. Consistent with previous evidence (13), the content-matched primary analysis showed no statistically significant difference between TR and face-to-face rehabilitation in terms of pain intensity; however, the certainty of the evidence was very low. The broader secondary analysis produced a similar estimate. The content-matched primary analysis similarly showed no statistically significant difference in functional disability between the two rehabilitation approaches. However, the pooled estimate was sensitive to the exclusion of one study, and the certainty of the evidence was low. In the exploratory subgroup analysis, the effect estimate for functional disability favored face-to-face rehabilitation in the unsupervised subgroup, whereas no statistically significant difference was observed in the supervised subgroup. However, because the interaction test was not statistically significant, the observed difference between subgroups does not provide evidence that supervision status modified the comparative effect. In the broader secondary analysis, the pooled estimate for functional disability favored face-to-face rehabilitation. Because this analysis included two trials in which adjunct manual therapy was provided exclusively to the face-to-face group, the pooled estimate may reflect differences in both delivery pathway and intervention composition and therefore cannot be attributed to delivery mode alone. By excluding paper-based home exercise programs, continuous hybrid delivery models, and remote-assessment-only designs, and by separating the content-matched primary analysis from the broader secondary analysis, this review provided a more interpretable comparison of the two rehabilitation pathways.

### 4.1 Comparison of effects on functional disability

The content-matched primary analysis showed no statistically significant difference between TR and face-to-face rehabilitation in functional disability, and the certainty of the evidence was low. Functional recovery is not determined by pain intensity alone and may also depend on progressive exercise, motor control, repeated movement practice, and the ability to perform daily activities (27–29). In the broader secondary analysis, which included two trials in which adjunct manual therapy was provided exclusively to the face-to-face group, the pooled estimate for functional disability favored face-to-face rehabilitation. The difference between the two analyses suggests that intervention composition may have contributed to the pooled estimate. Face-to-face rehabilitation may provide direct tactile correction, individualized therapist feedback, and adjunct manual therapy. These components could improve the quality of exercise performance and potentially confer a small functional benefit (30–32). The observed effect size was statistically small (SMD 0.26, 95% CI 0.01 to 0.52); however, its clinical importance remains uncertain. Because functional disability was pooled using standardized mean differences from different instruments, including the ODI and RMDQ, the pooled estimate could not be directly compared with established minimal clinically important difference thresholds (33). This finding also highlights an important difference between the two delivery pathways in clinical practice. Although TR may reduce geographic, time-related, and cost-related barriers, it generally cannot replicate hands-on palpation and manual therapy delivered by a therapist (34, 35). However, this interpretation remains speculative because the included studies did not directly examine whether differences in feedback or hands-on treatment produced clinically important differences in functional recovery. Further high-quality studies are therefore needed.

Exploratory subgroup analyses showed that the functional disability estimate favored face-to-face rehabilitation in the unsupervised subgroup, whereas no statistically significant difference was observed in the supervised subgroup. However, the test for subgroup interaction was not statistically significant, and the current evidence therefore does not support supervision status as an effect modifier. The observed pattern may inform future research, but it should be considered exploratory and confirmed in trials that directly compare supervised and unsupervised TR while holding intervention content constant.

The leave-one-out sensitivity analysis provided an additional reason for caution. After exclusion of Shi et al. (21), the pooled result changed from a nonsignificant difference to a statistically significant effect favoring face-to-face rehabilitation (SMD = 0.31, 95% CI 0.03 to 0.59), indicating that the statistical significance of the pooled estimate was sensitive to this study. In that trial, the TR group showed a greater mean improvement in ODI than the face-to-face group. The TR intervention integrated a smartphone application and sensors with automated reminders, exercise logging, and therapist monitoring and feedback. The TR group also had a slightly higher baseline ODI score and a higher intervention completion rate, which may have provided greater scope for improvement and supported patient engagement. However, the baseline ODI difference was not statistically significant, and intervention completion is not a direct measure of exercise adherence; these explanations therefore remain speculative. Previous evidence suggests that, among adults with chronic nonspecific low back pain, higher exercise adherence is associated with greater improvements in pain intensity and functional limitations, although the additional improvement relative to lower adherence is generally small and causality remains uncertain (36). Accordingly, this sensitivity analysis primarily indicates limited robustness of the pooled functional disability result and does not establish that any specific digital feature improves functional outcomes.

### 4.2 Comparison of effects on pain intensity

Neither the content-matched primary analysis nor the broader secondary analysis showed a statistically significant difference between TR and face-to-face rehabilitation in pain intensity. The similar pooled estimates may reflect the fact that both delivery pathways retained active exercise as the principal therapeutic component (21, 37). The inclusion of two trials in which adjunct manual therapy was provided exclusively to the face-to-face group did not alter the overall conclusion for pain intensity, with no statistically significant difference observed between the two rehabilitation approaches. Exercise-related pain improvement may arise through multiple interacting physiological mechanisms. Repeated movement and progressive loading may improve physical capacity and movement tolerance, reduce nociceptive input, facilitate endogenous pain modulation, and promote adaptive changes in central pain processing (38–41). However, the very low certainty of the evidence precludes firm conclusions that delivery mode has no influence on pain outcomes or that the two approaches have comparable effectiveness.

### 4.3 Strengths and limitations

A major strength of this review was the use of clearly defined eligibility criteria that excluded paper-based home exercise programs, continuous hybrid delivery models, and remote-assessment-only designs. By requiring a clearly identifiable delivery pathway during the core rehabilitation phase, the review reduced ambiguity when distinguishing TR from face-to-face rehabilitation. Separating the content-matched primary analysis from the broader secondary analysis also helped distinguish findings associated with the delivery pathway from those associated with intervention composition. Sensitivity analyses and exploratory subgroup analyses were used to examine the potential influence of individual studies, data conversion, intervention duration, weekly treatment frequency, and supervision status. Outcome-specific RoB 2 assessments and GRADE evaluations further improved the transparency of judgments regarding risk of bias and certainty of evidence.

Despite these strengths, several limitations should be considered. First, although the content-matched primary analysis minimized differences in intervention content, treatment frequency, dose, and duration, residual clinical heterogeneity remained across studies, particularly in supervision format and therapist contact inherent to the different delivery pathways. These differences may have influenced the pooled estimates and limited the extent to which observed effects could be attributed solely to the rehabilitation delivery mode. The small number of eligible studies and their limited sample sizes reduced statistical precision and restricted the reliability of the exploratory subgroup analyses. All assessed outcome results were judged to have some concerns regarding risk of bias, and the certainty of the evidence was very low for pain intensity and low for functional disability. In addition, the broader secondary analysis included two trials in which adjunct manual therapy was provided exclusively to the face-to-face group, making it difficult to attribute the pooled functional disability result to delivery mode alone. Because the meta-analysis pooled outcomes assessed at or near the end of the intervention, the long-term durability of treatment effects and differences in long-term effectiveness between telerehabilitation and face-to-face rehabilitation could not be evaluated. Some studies required the estimation of means or SDs from reported CIs, medians, or IQRs, which introduced additional statistical assumptions and uncertainty. However, excluding these studies did not materially alter the pooled estimates.

### 4.4 Implications for future research

Future trials should use standardized outcome definitions and transparently report therapist contact time, supervision intensity, exercise adherence, adherence-support strategies, and adverse events. Among the included studies, only one trial provided quantitative adherence data, reporting completion rates of 89% in the TR group and 81% in the face-to-face rehabilitation group after the 8-week intervention, with completion rates decreasing over time. The remaining studies mainly described adherence-support strategies without reporting quantitative adherence measures. Furthermore, adverse events were rarely reported, limiting conclusions regarding the safety of telerehabilitation. Future research should therefore prioritize standardized reporting of adherence and safety outcomes to better evaluate the feasibility and clinical implementation of telerehabilitation. Longer-term follow-up should also be incorporated to evaluate the durability of treatment effects and the risk of symptom recurrence. These improvements would help clarify which delivery pathway is most appropriate for particular patients, clinical settings, and stages of rehabilitation.

## 5 Conclusion

When the core rehabilitation intervention content was similar, no statistically significant differences were observed between TR and face-to-face rehabilitation as two rehabilitation delivery modes in either pain intensity or functional disability among adults with CLBP. However, given that the certainty of evidence was low for functional disability and very low for pain intensity, and that the functional disability estimate was sensitive to the exclusion of one study, these findings should not be interpreted as evidence of equivalent effectiveness between TR and face-to-face rehabilitation. In the broader secondary analysis, functional disability favored face-to-face rehabilitation. However, because this analysis included two trials in which adjunct manual therapy was provided exclusively to the face-to-face group, the result cannot be attributed to delivery mode alone and should be interpreted cautiously. Exploratory subgroup analyses did not provide evidence that intervention duration, weekly treatment frequency, or supervision status modified the comparative effect. Nevertheless, the differences observed in the analytical results between the supervised and unsupervised TR subgroups warrant attention in future trials.

Future research should use large, high-quality randomized controlled trials to compare TR with face-to-face rehabilitation while maintaining consistent core treatment content, treatment dose, and co-interventions. Longer follow-up periods are also needed to assess the maintenance of treatment effects and symptom recurrence. Direct comparisons of supervised and unsupervised TR are particularly needed. Future trials should transparently report therapist contact time, supervision intensity, exercise adherence, adherence-support strategies, and adverse events.

## Data Availability

The original contributions presented in the study are included in the article/Supplementary material, further inquiries can be directed to the corresponding authors.
