# Supplementary material for: Comparative effectiveness of telerehabilitation vs. face-to-face rehabilitation in adults with chronic low back pain: a systematic review and meta-analysis of randomized controlled trials
Source: Front Public Health. 2026 Sep 30;14:1965765. doi: 10.3389/fpubh.2026.1965765 (PMC13662063; doi:10.3389/fpubh.2026.1965765)
Supplement: Supplementary file 1 [file Data_Sheet_1.PDF]

PubMed:

#1:(((((((((((((((((((((((((((((Low Back Pain[MeSH Terms]) OR (Back Pain, Low[Title/Abstract])) OR (Back Pains, Low[Title/Abstract])) OR (Low Back Pains[Title/Abstract])) OR (Pain, Low Back[Title/Abstract])) OR (Pains, Low Back[Title/Abstract])) OR (Low Back Ache[Title/Abstract])) OR (Ache, Low Back[Title/Abstract])) OR (Aches, Low Back[Title/Abstract])) OR (Back Ache, Low[Title/Abstract])) OR (Back Aches, Low[Title/Abstract])) OR (Low Back Aches[Title/Abstract])) OR (Low Backache[Title/Abstract])) OR (Backache, Low[Title/Abstract])) OR (Backaches, Low[Title/Abstract])) OR (Low Backaches[Title/Abstract])) OR (Lower Back Pain[Title/Abstract])) OR (Back Pain, Lower[Title/Abstract])) OR (Back Pains, Lower[Title/Abstract])) OR (Lower Back Pains[Title/Abstract])) OR (Pain, Lower Back[Title/Abstract])) OR (Pains, Lower Back[Title/Abstract])) OR (Lumbago[Title/Abstract])) OR (Low Back Pain, Mechanical[Title/Abstract])) OR (Mechanical Low Back Pain[Title/Abstract])) OR (Low Back Pain, Posterior Compartment[Title/Abstract])) OR (Low Back Pain, Postural[Title/Abstract])) OR (Postural Low Back Pain[Title/Abstract])) OR (Low Back Pain, Recurrent[Title/Abstract])) OR (Recurrent Low Back Pain[Title/Abstract])

#2: ((((((((Telerehabilitation[MeSH Terms]) OR (Telerehabilitations[Title/Abstract])) OR (Tele-rehabilitation[Title/Abstract])) OR (Tele rehabilitation[Title/Abstract])) OR (Tele-rehabilitations[Title/Abstract])) OR (Remote Rehabilitation[Title/Abstract])) OR (Rehabilitation, Remote[Title/Abstract])) OR (Rehabilitations, Remote[Title/Abstract])) OR (Remote Rehabilitations[Title/Abstract])

#3: #1 AND #2

(((((((((((((((((((((((((((((Low Back Pain[MeSH Terms]) OR (Back Pain, Low[Title/Abstract])) OR (Back Pains, Low[Title/Abstract])) OR (Low Back Pains[Title/Abstract])) OR (Pain, Low Back[Title/Abstract])) OR (Pains, Low Back[Title/Abstract])) OR (Low Back Ache[Title/Abstract])) OR (Ache, Low Back[Title/Abstract])) OR (Aches, Low Back[Title/Abstract])) OR (Back Ache, Low[Title/Abstract])) OR (Back Aches, Low[Title/Abstract])) OR (Low Back Aches[Title/Abstract])) OR (Low Backache[Title/Abstract])) OR (Backache, Low[Title/Abstract])) OR (Backaches, Low[Title/Abstract])) OR (Low Backaches[Title/Abstract])) OR (Lower Back Pain[Title/Abstract])) OR (Back Pain, Lower[Title/Abstract])) OR (Back Pains, Lower[Title/Abstract])) OR (Lower Back Pains[Title/Abstract])) OR (Pain, Lower Back[Title/Abstract])) OR (Pains, Lower Back[Title/Abstract])) OR (Lumbago[Title/Abstract])) OR (Low Back Pain, Mechanical[Title/Abstract])) OR (Mechanical Low Back Pain[Title/Abstract])) OR (Low Back Pain, Posterior Compartment[Title/Abstract])) OR (Low Back Pain, Postural[Title/Abstract])) OR (Postural Low Back Pain[Title/Abstract])) OR (Low Back Pain, Recurrent[Title/Abstract])) OR (Recurrent Low Back Pain[Title/Abstract])

AND

((((((((Telerehabilitation[MeSH Terms]) OR (Telerehabilitations[Title/Abstract])) OR (Tele-rehabilitation[Title/Abstract])) OR (Tele rehabilitation[Title/Abstract])) OR (Tele-rehabilitations[Title/Abstract])) OR (Remote Rehabilitation[Title/Abstract]))

OR (Rehabilitation, Remote[Title/Abstract])) OR (Rehabilitations, Remote[Title/Abstract])) OR (Remote Rehabilitations[Title/Abstract])

Web of Science:

#1: (((((((((((ALL=(Low Back Pain)) OR ALL=(Low Back Pains)) OR ALL=(Low Back Ache)) OR ALL=(Low Back Aches)) OR ALL=(Low Backache)) OR ALL=(Low Backaches)) OR ALL=(Lower Back Pain)) OR ALL=(Lower Back Pains)) OR ALL=(Lumbago)) OR ALL=(Low Back Pain, Mechanical)) OR ALL=(Mechanical Low Back Pain)) OR ALL=(Postural Low Back Pain)) OR ALL=(Recurrent Low Back Pain)

#2: ((((((((ALL=(Telerehabilitation)) OR ALL=(Telerehabilitations)) OR ALL=(Tele-rehabilitation)) OR ALL=(Tele rehabilitation)) OR ALL=(Tele-rehabilitations)) OR ALL=(Remote Rehabilitation)) OR ALL=(Rehabilitation, Remote)) OR ALL=(Rehabilitations, Remote)) OR ALL=(Remote Rehabilitations)

#3: #1 AND #2

(((((((((((ALL=(Low Back Pain)) OR ALL=(Low Back Pains)) OR ALL=(Low Back Ache)) OR ALL=(Low Back Aches)) OR ALL=(Low Backache)) OR ALL=(Low Backaches)) OR ALL=(Lower Back Pain)) OR ALL=(Lower Back Pains)) OR ALL=(Lumbago)) OR ALL=(Low Back Pain, Mechanical)) OR ALL=(Mechanical Low Back Pain)) OR ALL=(Postural Low Back Pain)) OR ALL=(Recurrent Low Back Pain)

AND

((((((((ALL=(Telerehabilitation)) OR ALL=(Telerehabilitations)) OR ALL=(Tele-rehabilitation)) OR ALL=(Tele rehabilitation)) OR ALL=(Tele-rehabilitations)) OR ALL=(Remote Rehabilitation)) OR ALL=(Rehabilitation, Remote)) OR ALL=(Rehabilitations, Remote)) OR ALL=(Remote Rehabilitations)

Cochrane Library:

#1: Telerehabilitation

#2: Virtual Rehabilitations OR Rehabilitation, Remote OR Tele-rehabilitation OR Rehabilitations, Virtual OR Telerehabilitations OR Remote Rehabilitation OR Rehabilitation, Virtual OR Virtual Rehabilitation OR Tele rehabilitation OR Remote Rehabilitations OR Tele-rehabilitations OR Rehabilitations, Remote

#3: #1 OR #2

Telerehabilitation OR Virtual Rehabilitations OR Rehabilitation, Remote OR Tele-rehabilitation OR Rehabilitations, Virtual OR Telerehabilitations OR Remote Rehabilitation OR Rehabilitation, Virtual OR Virtual Rehabilitation OR Tele rehabilitation OR Remote Rehabilitations OR Tele-rehabilitations OR Rehabilitations, Remote

#4: Low Back Pain

#5: Low Back Pain, Recurrent OR Recurrent Low Back Pain OR Mechanical Low Back Pain OR Low Back Pain, Mechanical OR Back Pain, Low OR Pain, Lower Back OR Low Backache OR Back Ache, Low OR Lower Back Pain OR Pains, Low Back OR Backache, Low OR Back Pain, Lower OR Lumbago OR Backaches, Low OR Low Backaches OR Lower Back Pains OR Low Back Pains OR Back Pains, Lower OR Low Back Ache OR Aches, Low Back OR Ache, Low Back OR Back Pains, Low OR Pains, Lower Back OR Low Back Aches OR Pain, Low Back OR Back Aches, Low OR Low Back Pain, Posterior Compartment OR Low Back Pain, Postural OR Postural Low Back Pain

#6: #4 OR #5

Low Back Pain OR Low Back Pain, Recurrent OR Recurrent Low Back Pain OR Mechanical Low Back Pain OR Low Back Pain, Mechanical OR Back Pain, Low OR Pain, Lower Back OR Low Backache OR Back Ache, Low OR Lower Back Pain OR Pains, Low Back OR Backache, Low OR Back Pain, Lower OR Lumbago OR Backaches, Low OR Low Backaches OR Lower Back Pains OR Low Back Pains OR Back Pains, Lower OR Low Back Ache OR Aches, Low Back OR Ache, Low Back OR Back Pains, Low OR Pains, Lower Back OR Low Back Aches OR Pain, Low Back OR Back Aches, Low OR Low Back Pain, Posterior Compartment OR Low Back Pain, Postural OR Postural Low Back Pain

#7: #3 AND #6

Telerehabilitation OR Virtual Rehabilitations OR Rehabilitation, Remote OR Tele-rehabilitation OR Rehabilitations, Virtual OR Telerehabilitations OR Remote Rehabilitation OR Rehabilitation, Virtual OR Virtual Rehabilitation OR Tele rehabilitation OR Remote Rehabilitations OR Tele-rehabilitations OR Rehabilitations, Remote

AND

Low Back Pain OR Low Back Pain, Recurrent OR Recurrent Low Back Pain OR Mechanical Low Back Pain OR Low Back Pain, Mechanical OR Back Pain, Low OR Pain, Lower Back OR Low Backache OR Back Ache, Low OR Lower Back Pain OR Pains, Low Back OR Backache, Low OR Back Pain, Lower OR Lumbago OR Backaches, Low OR Low Backaches OR Lower Back Pains OR Low Back Pains OR Back Pains, Lower OR Low Back Ache OR Aches, Low Back OR Ache, Low Back OR Back Pains, Low OR Pains, Lower Back OR Low Back Aches OR Pain, Low Back OR Back Aches, Low OR Low Back Pain, Posterior Compartment OR Low Back Pain, Postural OR Postural Low Back Pain

Embase:

#1: telerehabilitation

#2: 'e-rehabilitation' OR 'remote rehabilitation' OR 'tele-rehabilitation' OR 'virtual rehabilitation' OR 'telerehabilitation'

#3: #1 OR #2

telerehabilitation OR 'e-rehabilitation' OR 'remote rehabilitation' OR 'tele-rehabilitation' OR 'virtual rehabilitation' OR 'telerehabilitation'

#4: low back pain

#5: 'acute low back pain' OR 'back pain, low' OR 'chronic low back pain' OR 'loin pain' OR 'low backache' OR 'low backpain' OR 'lowback pain' OR 'lower back pain' OR 'lumbago' OR 'lumbal pain' OR 'lumbal syndrome' OR 'lumbalgesia' OR 'lumbalgia' OR 'lumbar pain' OR 'lumbar spine syndrome' OR 'lumbodynia' OR 'lumbosacral pain' OR 'lumbosacral root syndrome' OR 'lumbosacroiliac strain' OR 'pain, low back' OR 'pain, lumbosacral' OR 'strain, lumbosacroiliac' OR 'low back pain'

#6: #4 OR #5

low back pain OR 'acute low back pain' OR 'back pain, low' OR 'chronic low back pain' OR 'loin pain' OR 'low backache' OR 'low backpain' OR 'lowback pain' OR 'lower back pain' OR 'lumbago' OR 'lumbal pain' OR 'lumbal syndrome' OR 'lumbalgesia' OR 'lumbalgia' OR 'lumbar pain' OR 'lumbar spine syndrome' OR 'lumbodynia' OR 'lumbosacral pain' OR 'lumbosacral root syndrome' OR 'lumbosacroiliac strain' OR 'pain, low back' OR 'pain, lumbosacral' OR 'strain, lumbosacroiliac' OR 'low back pain'

#7: #3 AND #6

telerehabilitation OR 'e-rehabilitation' OR 'remote rehabilitation' OR 'tele-rehabilitation' OR 'virtual rehabilitation' OR 'telerehabilitation'

AND

low back pain OR 'acute low back pain' OR 'back pain, low' OR 'chronic low back pain' OR 'loin pain' OR 'low backache' OR 'low backpain' OR 'lowback pain' OR 'lower back pain' OR 'lumbago' OR 'lumbal pain' OR 'lumbal syndrome' OR 'lumbalgesia' OR 'lumbalgia' OR 'lumbar pain' OR 'lumbar spine syndrome' OR 'lumbodynia' OR 'lumbosacral pain' OR 'lumbosacral root syndrome' OR 'lumbosacroiliac strain' OR 'pain, low back' OR 'pain, lumbosacral' OR 'strain, lumbosacroiliac' OR 'low back pain'
