# Supplementary material for: Comparative effectiveness of telerehabilitation vs. face-to-face rehabilitation in adults with chronic low back pain: a systematic review and meta-analysis of randomized controlled trials
Source: Front Public Health. 2026 Sep 30;14:1965765. doi: 10.3389/fpubh.2026.1965765 (PMC13662063; doi:10.3389/fpubh.2026.1965765)
Supplement: Supplementary file 2 [file Supplementary_file_1.DOCX]

**Figure S1.** Funnel plot for the secondary analysis of functional disability. The x-axis represents the standardized mean difference (SMD), and the y-axis represents the standard error (SE). The dashed lines indicate the pseudo 95% confidence limits, and the solid vertical line represents the pooled effect estimate.

**Figure S2.** Results of sensitivity analyses for the primary outcomes. (a) Leave-one-out sensitivity analysis for functional disability. (b) Sensitivity analysis for functional disability after excluding studies in which means or SDs were estimated from reported 95% CIs, medians, or IQRs. (c) Sensitivity analysis for pain intensity after excluding studies in which means or SDs were estimated from reported 95% CIs, medians, or IQRs.
