# Supplementary material for: Comparative effectiveness of telerehabilitation vs. face-to-face rehabilitation in adults with chronic low back pain: a systematic review and meta-analysis of randomized controlled trials
Source: Front Public Health. 2026 Sep 30;14:1965765. doi: 10.3389/fpubh.2026.1965765 (PMC13662063; doi:10.3389/fpubh.2026.1965765)
Supplement: Supplementary file 3 [file Table_3.DOCX]

**Table S3. Summary of results and evaluation of the certainty of evidence (GRADE).**

| **Quality assessment** | | | | | | | **No of patients** | | **Effect** | | **Quality** | **Importance** |
| --- | --- | --- | --- | --- | --- | --- | --- | --- | --- | --- | --- | --- |
|  |  |  |  |  |  |  |  |  |  |  |  |  |
| **No of studies** | **Design** | **Risk of bias** | **Inconsistency** | **Indirectness** | **Imprecision** | **Other considerations** | **Telerehabilitation Group** | **Face-to-face Group** | **Relative (95% CI)** | **Absolute** |  |  |
| **functional limitation** (Lower values indicate better efficacy in the telerehabilitation group) | | | | | | | | | | | | |
| 8 | randomised trials | serious^1^ | no serious inconsistency | serious^2^ | no serious imprecision | none | 282 | 280 | - | SMD 0.22 higher (0.07 lower to 0.52 higher) | ⊕⊕OO LOW | CRITICAL |
| **Pain intensity (**Lower values indicate better efficacy in the telerehabilitation group**)** | | | | | | | | | | | | |
| 7 | randomised trials | serious^1^ | no serious inconsistency | serious^2^ | serious^3^ | none | 205 | 209 | - | SMD 0.05 lower (0.34 lower to 0.24 higher) | ⊕OOO VERY LOW | CRITICAL |

^1^ Downgraded one level for risk of bias because all included studies were judged as having some concerns in the outcome-specific RoB 2 assessment.
^2^ Downgraded one level for inconsistency/indirectness due to heterogeneity in interventions, supervision, co-interventions, and outcome measures.

^3^ Downgraded one level for imprecision because the confidence interval crossed the line of no effect and the total sample size was small.
